# Effect of immunosuppressive medication on postoperative complications following abdominal surgery in Crohn’s disease patients

**DOI:** 10.1007/s00384-022-04287-4

**Published:** 2022-11-28

**Authors:** Saleh Lahes, Celine Fischer, Antonios E. Spiliotis, Antje Schulz, Gereon Gäbelein, Dorian Igna, Matthias Glanemann

**Affiliations:** Department of General, Visceral, Vascular and Pediatric Surgery, University of Saarland, Kirrberger Straße, 66421 Homburg, Saarland Germany

**Keywords:** Crohn’s disease, Immunosuppressive agents, Colorectal surgery, Postoperative complications

## Abstract

**Background:**

Immunosuppressants represent an effective pharmacological treatment for the remission and management of Crohn’s disease (CD); however, it has not been well-defined if these medications are associated with an increased incidence of postoperative complications after intestinal surgery. This retrospective study evaluated the association between immunosuppressive treatment and complications following bowel resection in patients with CD.

**Methods:**

A total of 426 patients with CD who underwent abdominal surgery between 2001 and 2018 were included in the study. The participants were divided into two groups. In the first group, patients were under immunosuppressive treatment at the time of surgical resection, while in the second group, patients had never received pharmacological therapy for CD before surgery.

**Results:**

No statistically significant difference was found in the incidence of postoperative complications between the two groups. Double or triple immunosuppressive therapy was not associated with increased complications compared to monotherapy or no pharmacological treatment. Preoperative risk factors such as hypoalbuminemia, abscess, fistula, intestinal perforation, long duration of symptoms, and the intraoperative performance of more than one anastomosis were related to increased rates of postoperative complications. Factors affecting the occurrence of postoperative complications in the univariate analysis were included in the multivariate analysis using a stepwise logistic regression model, and these factors were also related to increased rates of postoperative surgical complications.

**Conclusion:**

Immunosuppressive therapy was not associated with increased rates of postoperative complications following bowel resection in patients with CD.

## Introduction

Crohn’s disease (CD) is an idiopathic, chronic, and transmural inflammatory disease of unknown etiology that can affect any segment of the gastrointestinal tract from the mouth to the anus.

Most cases involve the terminal ileum and coecum, and the highest incidence has been reported in individuals during their third decade of life [1, 2].

Pharmacological management is recommended as the first-line treatment for patients with an uncomplicated disease. In contrast, surgical treatment is indicated when medical therapy is unsuccessful in disease remission or in cases of CD-related complications [3]. Several complications can occur during the course of the disease, including fistula, abdominal abscess, intestinal perforation, stenosis, or obstruction. The most frequent complication is acute intestinal obstruction, which occurs in 35–54% of cases located in the terminal ileum and ileocecal junction [4]. Free intestinal perforation is reported in 1–3% of patients and is an indication that emergency surgery is required [5]. The incidence of intra-abdominal abscess is estimated to be 10–30% and is associated with fistula and intestinal stenosis in 40% and 51% of patients, respectively [6]. Approximately 70–90% of patients with CD undergo surgical treatment during their lifetime, namely ileocolectomy in the majority of cases [7–10].

An increased complication rate, principally anastomotic complications, intra-abdominal septic complications, and wound infections are reported after bowel resection in patients with CD compared to patients without an inflammatory disease [11].

Immunosuppressive agents are considered potential causal factors for increased postoperative morbidity. Most patients undergoing surgery for Crohn’s disease are treated with immunomodulatory agents, either as a single medication or as a combined treatment (including steroids, immunosuppressants, or biologic agents). The effect of immunosuppressive medications on surgical outcomes is debatable.

Although some studies have reported that immunosuppressive medications are associated with increased postoperative complications [12–14], others have failed to demonstrate a relationship between immunosuppressants and complicated postoperative courses [15–18].

This retrospective study was conducted with the aim to evaluate the role of immunosuppressive regimens in the rate of postoperative complications following bowel resection surgery in patients with CD.

## Methods

### Study design and participants

All patients who underwent abdominal surgery for CD between 2001 and 2018 in the Department of General Surgery, Visceral, Vascular, and Pediatric Surgery at the University Hospital of Saarland were identified and evaluated for inclusion in the study. Only histologically confirmed CD cases were included in the analysis. A total of 426 patients who underwent small bowel or colorectal resection, ileostomy or colostomy, closure of ileostomy or colostomy, or strictureplasty were included in the study. Other types of surgical procedures without bowel resection or anastomosis, such as surgical drainage of intraabdominal abscess, fistula closure, and adhesiolysis, were excluded from the analysis.

Demographics and relevant perioperative data were extracted from patient charts, including age, sex, body mass index (BMI), disease duration, history of previous surgical procedures, type of medical treatment for CD, indication for surgery, elective or emergency surgery, type of surgical procedure, and preoperative albumin level.

A complicated course of CD was defined by the presence of an intra-abdominal abscess, fistula (enterovesical, enterovaginal, enterocutaneous, or enteroenteric), or bowel perforation as an indication for abdominal surgery. Other indications for abdominal surgery, such as bowel obstruction, intestinal stricture, medication-refractory disease, or elective closure of a stoma, were also included in the study.

Treatment protocols with mesalazine, corticosteroids, immunomodulators (azathioprine, 6-mercaptopurine), or anti-TNF-α antibodies have been documented.

The patients were divided into two groups. The first group consisted of patients who received immunosuppressive medication at the time of surgery or just before. Patients who had never received pharmacological treatment prior to surgery were included in the second group.

### Primary and secondary outcomes

The primary outcome was the incidence of surgical complications. Thirty-day morbidity and mortality rates following intestinal surgery for CD were examined in this study.

Anastomotic leak, stoma retraction or necrosis, wound infection, intra-abdominal abscess, fistula, bleeding, peritonitis, sepsis following surgical complication, and reoperation rate were included as primary endpoints. Thirty-day mortality included patients who died in the hospital, at home after discharge, and those transferred to other care facilities or in a convalescent environment. Patients with anastomotic leaks who were treated conservatively, with intervention (vacuum-assisted closure therapy or image-guided percutaneous drainage), or surgically were included in the study. Stoma retraction or necrosis, abscesses, and fistulas were treated conservatively or surgically. Patients with wound infections treated at the bedside or in the operating theater were included in the analysis. Postoperative bleeding was defined as any intra-abdominal hemorrhage that was treated with or without transfusion, interventional radiology, or surgery. Abscess and fistula were treated conservatively or surgically.

Postoperative pulmonary complications, included in the secondary outcomes, were pneumonia, pleural effusion, pleural empyema, pneumothorax, pulmonary edema, atelectasis, acute respiratory distress syndrome, and respiratory insufficiency. Cardiac complications (cardiac arrest, myocardial infarction, atrial or ventricular dysrhythmia requiring treatment), urological complications (acute renal insufficiency, urinary tract infection), thromboembolic complications (deep venous thrombosis and pulmonary embolism), and postoperative delirium were included as secondary outcomes of our study.

### Statistical analysis

Statistical analysis was performed using the SPSS® version 20.0 software (IBM, Armonk, NY, USA). Categorical data were presented as absolute numbers and percentages. Statistical analysis was performed using Pearson’s chi-squared test. Univariate analysis of the effects of selected predictive factors on postoperative complications was performed using the Pearson chi-square test. Factors affecting the occurrence of postoperative complications in the univariate analysis were included in the multivariate analysis using a stepwise logistic regression model, and the results are presented as *p*-values. All *p*-values were 2-sided. A *p*-value equal to or less than 0.05 is considered significant.

## Results

### Patient characteristics

A total of 426 patients (189 men, 237 women) were included in the study. Indications for surgery were a medication-refractory disease; CD-related complications, such as intestinal perforation, intestinal stricture, intraabdominal abscess, or fistula; and elective closure of an ileostomy or colostomy. A total of 158 patients had more than a sole indication for surgery. Table 1 provides an overview of the procedures performed in all groups. The most common operative procedure was ileocecal resection in 46% of patients, followed by colic resection with anastomosis or stoma (17.1%), closure of an ileostomy or colostomy (10.6%), and total colectomy (9.2%). The median age was 41 years (range, 16–73 years).

**Table 1:** Type of operative procedures

**Operative procedure**	**Group of patients**		
	**Group 1** ***n* = 329 (77.2%)**	**Group 2** ***n* = 97 (22.8%)**	**Total** ***n* = 426**
**Ileocecal resection**	155 (36.4%)	41 (9.6%)	196 (46%)
**Colon resection**	68 (15.9%)	5 (1.2%)	73 (17.1%)
**Closure of ileostomy or colostomy**	31 (7.3%)	14 (3.3%)	45 (10.6%)
**Total colectomy**	32 (7.5%)	7 (1.7%)	39 (9.2%)
**Ileostomy or colostomy**	18 (4.2%)	13 (3%)	31 (7.3%)
**Proctocolectomy**	16 (3.8%)	8 (1.9%)	24 (5.6%)
**Small intestine resection**	9 (2.1%)	8 (1.9%)	17 (4%)
**Strictureplasty**	0	1 (0.2%)	1 (0.2%)

### Postoperative complications

The incidence rates of overall complications and surgical complications were 37.1% and 28.6%, respectively. Eighty-eight patients presented with one postoperative surgical complication, whereas 34 patients had more than one. The most common surgical complication was wound infection (21.4%), followed by anastomotic leak (7.3%) and intra-abdominal abscess (5.4%).

Table 2 illustrates the surgical complications in each patient group. Postoperative surgical complications, such as wound infection, fascia dehiscence, intra-abdominal abscess, anastomotic leakage, internal fistula, and bleeding, were documented.

**Table 2:** Type of postoperative complications in the three groups of patients

	Group 1 329 patients	Group 2 97 patients	*p*-value	Total
Incidence of surgical complications	96 (29.2%)	26 (26.8%)	0.649	122 (28.6%)
Wound infection	72 (21.9%)	19 (19.6%)	0.628	91 (21.4%)
Anastomotic leak	26 (7.9%)	5 (5.2%)	0.36	31 (7.3%)
Abscess	18 (5.5%)	5 (5.2%)	0.904	23 (5.4%)
Peritonitis	10 (3%)	1 (1%)	0.273	11 (2.6%)
Fistula	9 (2.9%)	2 (2.1%)	0.713	11 (2.6%)
Bleeding	5 (1.6%)	5 (5.2%)	0.038	10 (2.3%)
Sepsis	10 (3%)	0 (0%)	0.082	10 (2.3%)
30-day mortality	6 (1.8%)	0 (0%)	0.18	6 (1.4%)
Stoma retraction/necrosis	5 (1.6%)	0 (0%)	0.222	5 (1.2%)
Reoperation	73 (22.2%)	22 (22.7%)	0.919	95 (22.3%)
Incidence Non surgical complication	58 (17.6%)	15 (15.5%)	0.619	73 (17.1%)

The Pearson chi-square test was used. Categorical data were presented as absolute numbers and percentages

Anastomotic leaks were also reported in 31 patients. Specifically, anastomotic leaks were detected in 7.9% and 5.2% of patients in groups 1 and 2, respectively. No significant differences were found between the groups (*p* = 0.36). Surgical treatment for the anastomotic leak is required in more than two-thirds of the cases. Overall, the 30-day mortality rate was 1.4%. Although mortality was reported in only six patients in group 1, a significant difference between the groups was not detected. Mortality is attributed to pneumonia, sepsis, and multiple organ failure. The incidence of other surgical complications, including peritonitis, stoma retraction/necrosis, bleeding, abscess, fistula, wound infection, and reoperations, was comparable between the groups. Furthermore, no significant differences were observed in the incidence of secondary complications.

As shown in Table 3, specific preoperative and intraoperative parameters were examined as risk factors for surgical and overall complications in univariate analysis. Age and BMI were not associated with increased postoperative complication rates.

**Table 3:** Relationship between the severity of postoperative complications and preoperative parameters

**Variables**	**Number of patients, *n***	**Univariate analysis**				**Multivariate analysis**	
		**Postoperative complications**				**Surgical overall**	
		**Surgical (%)**		**Overall (%)**			
		*n* (%)	*p*-value	*n* (%)	*p*-value	*p*-value	*p*-value
**Overall collective**	**426**	122 (28.6%)		158 (37.1%)			
**Age, years**			0.182		0.06		
< 20	13	4 (30.8%)		4 (30.8%)			
20–50	328	87 (26.5%)		113 (34.5%)			
> 50	85	31 (36.5%)		41 (48.2%)			
**Duration of symptoms, years**	426		0.009		0.017	0.043	0.903
< 1	39	7 (17.9%)		11 (28.2%)			
1–10	177	41 (23.2%)		55 (31.1%)			
> 10	210	74 (35.2%)		92 (43.8%)			
**Number of previous operations**	426		0.051		0.126		
0	104	20 (19.2%)		31 (29.8%)			
1	121	39 (32.2%)		52 (43%)			
≥ 2	201	63 (31.3%)		75 (37.3%)			
**BMI, kg/m**^**2**^			0.623		0.756		
≤ 18,4	57	15 (26.3%)		20 (35.1%)			
18.5–24.9	230	63 (27.4%)		83 (36.1%)			
≥ 25	139	44 (31.7%)		55 (39.6%)			
**Group of patients**			0.649		0.636		
Group 1	329	96 (29.2%)		124 (37.7%)			
Group 2	97	26 (26.8%)		34 (35.1%)			
**Type of surgical procedure** Emergency Elective	58 368	19 (32.8%) 103 (28%)	0.532	25 (43.1%) 133 (36.1%)	0.380		
**Preoperative complications**			< 0.001		< 0.001	< 0.001	0.013
Yes	193	81 (42%)		96 (49.7%)			
No	233	41 (17.6%)		62 (26.6%)			
** Abscess**	50	21 (42%)	0.031	22 (44%)	0.350		
** Internal fistula**	142	61 (43%)	< 0.001	73 (51.4%)	< 0.001		
**Perforation**	34	16 (47.1%)	0.017	19 (55.9%)	0.025		
**Anastomoses**			< 0.001		< 0.001	0.026	0.860
Yes	339	83 (24.5%)		111 (32.7%)			
No	87	39 (44.8%)		47 (54%)			
**Number of anastomoses**			0.001		0.002		
1	321	77 (24.1%)		104 (32.5%)			
2	17	5 (29.4%)		6 (35.3%)			
≥ 3	1	1 (50%)		1 (50%)			
**Location of anastomosis**							
Small/small bowel	43	14 (32.6%)	0.594	19 (44.2%)	0.321		
Ileocolic	259	61 (23.5%)	0.004	80 (30.8%)	0.001		
Colocolic	13	4 (30.8%)	1.000	4 (30.8%)	0.775		
Ileorectal	19	4 (21.1%)	0.606	8 (42.1%)	0.809		
Colorectal	25	7 (28%)	1.000	8 (32%)	0.673		
**Serum albumin**	251	78 (31.1%)	0.003	96 (38.2%)	0.002		
< 30 mg/dl	31	17 (54.8%)		20 (64.5%)			
≥ 30 mg/dl	220	61 (27.7%)		76 (34.5%)			

Univariate analysis was performed using the Pearson chi-square test and multivariate analysis using a stepwise logistic regression model. Categorical data were presented as absolute numbers and percentages

A significant correlation was found between disease duration and postoperative complications. Specifically, patients with a long history of CD present with increased rates of complications compared to those with a short course of disease.

Furthermore, according to our results, patients with a history of previous abdominal surgeries tended to have increased postoperative surgical complications compared to those with no history of previous surgery (*p* = 0.051).

Approximately seventy-seven of the patients had received immunosuppressive medication at the time of surgery or just before, whereas in all other cases, patients had never received immunosuppressive agents. Statistical analysis showed no difference in the incidence of complications among the two groups, indicating that immunosuppression was not related to an increased complication rate (Table 3). Both groups presented a rate of surgical complications estimated at 26.8–29.2%.

Furthermore, an overall analysis was conducted comparing the patients under an immunosuppressive regimen from group 1 with those from group 2 without immunosuppressive therapy at the time of surgery. The percentage of surgical complications was 29.2% in group 1 and 26.8% in group 2 (*p* = 0.649). Similarly, no significant difference was found in the incidence of overall complications (*p* = 0.636).

Emergency surgery was required in 58 patients, while 368 patients underwent elective surgery.

No significant difference in the incidence of postoperative complications was found between elective and emergency surgeries.

In patients with preoperative CD-related complications, such as intra-abdominal abscess, internal fistula, or perforation, increased rates of surgical and overall complications were observed compared to those in patients with no relevant preoperative morbidity (*p* < 0.001).

Surgical resection with intestinal anastomosis was performed in 339 cases, whereas anastomosis was not possible in 87 cases because of septic shock, peritonitis, or other reasons related to a high risk of anastomotic failure. The incidence of complications was significantly decreased in the group of patients who underwent anastomosis (*p* < 0.001).

Preoperative serum albumin levels were recorded for 251 patients. A low albumin level (< 30 mg/dl) was found in 31 patients and was associated with a significantly higher incidence of surgical and overall complications.

The parameters associated with an increased incidence of surgical complications in the univariate analysis were included in the multivariate analysis using a stepwise logistic regression model. Albumin and the number of anastomoses were excluded from the multivariate analysis because albumin levels were not available for all participants, and anastomosis was not conducted in all cases. In multivariate analysis, a complicated course of CD, absence of anastomosis, and long duration of CD were significantly associated with increased surgical complications.

The incidence of complications was compared between different types of immunosuppressive and anti-inflammatory medications among patients undergoing pharmacological therapy. As shown in Table 4, no differences were found in complications between the patients treated with different anti-inflammatory and immunosuppressant agents.

**Table 4:** Type of anti-inflammatory and immunosuppressive medication and postoperative complications

**Variables**	***n***	**Postoperative complications**			
		**Surgical complications (%)**		**Overall complications (%)**	
	**426**	*n* (%)	*p*-value	*n* (%)	*p*-value
**Immunosuppressive therapy** Yes None	**329** **97**	96 (29.2%) 26 (26.8%)	0.649	124 (37.7%) 34 (35.1%)	0.636
**Steroids**	**94**	28 (39.8%)	0.780	39 (41.5%)	0.317
**Salicylates**	**28**	5 (15%)	0.192	8 (28.6%)	0.334
**Immunomodulators**	**20**	3 (16.7%)	0.167	5 (25%)	0.252
**Anti-TNF**	**38**	11 (28.9%)	0.965	13 (34.2%)	0.700
**Salicylates + others**	**91**	30 (33%)	0.303	39 (42.9%)	0.199
**Steroids + immunomodulators**	**25**	10 (40%)	0.195	10 (40%)	0.756
**Anti-TNF + Steroids**	**19**	5 (26.3%)	0.819	6 (31.6%)	0.611
**Anti-TNF + immunomodulators**	**9**	3 (33.3%)	0.753	3 (33.3%)	0.814
**Anti-TNF + steroids + immunomodulators**	**5**	1 (20%)	0.667	1 (20%)	0.426

Pearson chi-square test was used. Categorical data were presented as absolute numbers and percentages

## Discussion

Immunosuppressive agents are fundamental for the remission and management of CD; however, current evidence in the literature regarding their association with postoperative complications is controversial. We conducted a retrospective study to evaluate the incidence of surgical and overall complications in patients who had received or never received immunosuppressants at the time of surgery. A total of 426 patients were included in this retrospective analysis.

Most patients (77.2%) had received immunosuppressants at the time of surgery or just before, and 29.2% developed at least one postoperative surgical complication. In contrast, 22.8% of our patients had never received immunosuppressive medication, and in this group, the incidence of postoperative surgical complications was as high as that in those with continuous immunosuppressants, estimated at 26.8% and 29.2%, respectively.

In our analysis, perioperative immunosuppressive therapy was not significantly associated with an increased rate of postoperative complications. In accordance with our results, a retrospective study conducted by Indar et al. with 112 patients reported that 32% and 26% of patients with and without immunosuppressive therapy developed postoperative complications, respectively [18]. No significant differences were observed. Mascarenhas et al. evaluated the role of anti-TNF agents in the incidence of postoperative complications [19]. Although the rate of major complications increased among patients under treatment with anti-TNF medications, this difference was not statistically significant (CD with biologics: 10.5% vs. CD without biologics: 4.1%).

Two hundred and twenty-five patients were included in a retrospective study conducted by Canedo and colleagues and were divided into the infliximab group, steroids and/or immunosuppressants group, and no pharmacologically treated group [20]. There was no difference in the rate of postoperative complications between the three groups.

A retrospective study with a total of 166 patients, of whom only 28 patients used steroids for a period of 3 months or more before surgery, showed that 29% of those patients developed intra-abdominal septic complications [21]. Although the univariate analysis showed a significant association between steroid use and septic complications, the multivariate analysis failed to provide a significant result. Shental et al. concluded that this result could be attributed to a type II error due to the limited number of patients and mentioned the need for a study with more participants.

A meta-analysis of 1159 patients examined the complication rate in patients treated with infliximab, an anti-TNF-α blocker [22]. There was no significant difference in the rates of major and minor complications, reoperations, and 30-day mortality between the infliximab and control groups. An association between anti-TNF agents or steroids and overall complications was not found in a retrospective study of 538 patients from two specialist centers in the Netherlands and Belgium. More than 250 patients were treated with anti-TNF agents or steroids, and the overall morbidity rate was estimated at 22.5% [23]. In accordance with our results, a multicenter study of 231 patients from seven referral centers in Japan, Brazil, and Italy concluded that preoperative immunosuppression is not a risk factor for complications [24]. Of the patients treated with steroids, immunosuppressants, and biologics, 26%, 22%, and 22%, respectively, developed postoperative complications.

In contrast, some studies have reported a significant association [14]. Post et al. found a significant association between steroid use and postoperative complications.

A meta-analysis of 21 included studies concluded that patients receiving monotherapy with anti-TNF agents or corticosteroids were at a higher risk of postoperative infectious complications. There was no association between the use of thiopurines or immunomodulatory drugs and postoperative complications [25]. However, the authors highlighted the heterogeneity of the assessed data and reported suboptimal quality of the included studies.

Age and BMI did not play a significant role in assessing risk factors for postoperative complications. By contrast, we found that patients with a long history of CD had an increased rate of postoperative complications. In many cases, a prolonged disease course is associated with advanced medication-refractory disease and severe bowel inflammation, which could deteriorate the postoperative course. Furthermore, patients with a history of one or more abdominal surgeries had a high risk of postoperative complications, although the results were not statistically significant. A retrospective study with 621 patients found in univariate and multivariate analysis that previous intestinal resections constitute a significant factor that increases the risk for postoperative complications by 11% [26].

Most patients underwent surgical resection with anastomosis, whereas in cases of peritonitis, sepsis, or other reasons related to a high risk of anastomotic failure, surgical resection with stoma was performed. Patients without anastomosis showed increased postoperative complications, which could be attributed to their critical status at the time of surgery. Furthermore, we found that patients with two or more anastomoses experienced significantly more complications than those with one anastomosis. In contrast to our results, other studies have reported a similar incidence of complications regardless of the number of anastomoses [14, 27].

Preoperative complications of CD, such as abscess, fistula, or intestinal perforation, were associated with increased postoperative complications. Several studies have found that the presence of an intra-abdominal abscess or fistula at the time of surgery for CD increases the risk of postoperative intra-abdominal septic complications [27–29]. In contrast, other studies have found no association between preoperative abscess or fistula and postoperative intra-abdominal septic complications [14, 21, 22, 30, 31].

Serum albumin accelerates wound healing and collagen synthesis at the anastomosis site. Hypoproteinemia is associated with tissue edema and collagen synthesis disorders, which may contribute to anastomotic leakage [32]. In our study, a low preoperative albumin level was associated with an increased incidence of postoperative complications. In agreement with these results, a study conducted by Yamamoto et al. reported a significant association between low albumin levels and complications in univariate (*p* = 0.01) and multivariate analyses (*p* = 0.04) [27]. Correction of severe hypoalbuminemia is necessary during the perioperative management.

As with the majority of studies, the design of the current study is subject to limitations, namely a retrospective design and a selection bias leading to only limited definitive conclusions. The patients were not randomized into the two treatment groups. Although our study included a large number of patients, hidden bias is inevitable because additional parameters, which may influence the results, were not taken into account. However, the study design, collective results of all patients with CD, and large number of patients provided safe evidence regarding the association between immunosuppressive agents and postoperative complications. To the best of our knowledge, this retrospective study represents the most contemporary analysis of a large number of patients with CD that has evaluated the role of immunosuppressive therapy in the postoperative course.

In the literature, the postoperative complication rates in patients with CD are high. Despite the fact that modern immunosuppressive drugs are currently able to better manage the symptoms of Crohn’s disease, the incidence of operative therapy is also high and remains the therapy of choice in some cases [33]. It is imperative that the treatment of patients with CD be interdisciplinary.

## Conclusion

Our results showed that the use of immunosuppressive medications was not associated with an increased incidence of postoperative complications. Other risk factors such as low preoperative albumin level, preoperative intra-abdominal complications, long disease duration, and the number of anastomoses were correlated with an increased incidence of complications.

## Data Availability

The datasets generated during and/or analyzed during the current study are not publicly available, but are available from the corresponding author on reasonable request.
